# Association of life’s essential 8 with chronic obstructive pulmonary disease: a population-based analysis of NHANES 2007–2018

**DOI:** 10.1186/s12889-024-20534-5

**Published:** 2024-11-13

**Authors:** Yuhang Liu, Weidong Li, Jialing Tang, Siyao Gao

**Affiliations:** 1 School of Physical Education and Sports, Central China Normal University, Wuhan, 430079 P.R. China; 2 Department of Physical Education, Central South University, No. 932 Lushan South Road, Yuelu District, Changsha, 410083 P.R. China

**Keywords:** Life’s essential 8, Cardiovascular health, Chronic obstructive pulmonary disease, NHANES

## Abstract

**Background:**

Chronic obstructive pulmonary disease (COPD) is closely linked to cardiovascular diseases. We aimed to investigate the association between Life’s Essential 8 (LE8), the newly established measurement to assess cardiovascular health (CVH), and COPD among U.S. general adults.

**Methods:**

This study extracted the National Health and Nutrition Examination Survey (2007–2018) data. Multivariate logistic regression models were used to examine the associations between LE8 and COPD. A restricted cubic spline regression model was used to explore the dose-response relationships between LE8 scores and COPD. In addition, subgroup and sensitivity analyses were performed to assess the robustness of our results.

**Results:**

Our study included 19,774 participants representing 145.2 million non-institutionalized U.S. population aged ≥ 20 years. The overall age-adjusted prevalence of COPD was 4.5%. After adjusting for the potential covariates, LE8 was inversely associated with COPD [adjusted odds ratio (AOR) = 0.169, 95% CI: 0.115, 0.249], exhibiting a nonlinear dose-response relationship (*P* for nonlinearity < 0.05). Similar trends in the associations of health behavior score (AOR = 0.300, 95% CI: 0.223, 0.404) and health factor score (AOR = 0.603, 95% CI: 0.426, 0.852) with COPD were also identified. Furthermore, higher LE8 metric scores of nicotine exposure and sleep health were associated with a lower prevalence of COPD.

**Conclusion:**

LE8 was inversely associated with spirometric/self-reported COPD in a nonlinear trend, primarily driven by the nicotine exposure metric of LE8. Adhering to LE8 guidelines, especially smoking cessation, to sustain optimal CVH levels may be beneficial to alleviate the burden of COPD.

**Supplementary Information:**

The online version contains supplementary material available at 10.1186/s12889-024-20534-5.

## Introduction

Chronic obstructive pulmonary disease (COPD) is a heterogeneous lung condition, characterized by chronic respiratory symptoms (dyspnea, cough, expectoration and/or exacerbations) due to abnormalities of the airways (bronchitis, bronchiolitis) and/or alveoli (emphysema) that cause persistent, often progressive, airflow obstruction [1]. According to the World Health Organization, COPD, the third leading cause of death worldwide, kills more than 3 million people worldwide every year [2]. It is estimated to affect over 330 million individuals globally [3]. In addition, the association of COPD with cardiovascular diseases (CVD) is well-recorded [4]. A large body of evidence suggests that COPD patients have an elevated risk of developing CVD compared with the non-COPD population [4–6]. Moreover, cardiovascular mortality was higher in patients with reduced pulmonary function [7, 8].

In 2022, the American Heart Association (AHA), based on Life’s Simple 7 (LS7), established a novel and measurable indicator called Life’s Essential 8 (LE8) to assess and promote cardiovascular health (CVH) in the general population [9, 10]. It consists of four health behaviors [diet, physical activity (PA), nicotine exposure, and sleep health] and four health factors [body mass index (BMI), total cholesterol, blood pressure (BP), and blood glucose]. Relative to LS7, LE8 adds the sleep health metric and is more sensitive to differences between and within individuals. In addition, based on LE8 scores, the individual’s CVH was classified as low, moderate, or high 3 levels. The latest studies have demonstrated that a higher LE8 score is associated with several health outcomes and indicates healthier behaviors. For instance, Sun et al. reported that a higher LE8 score was linked to a reduced risk of all-cause and CVD-specific mortality [11]. Ma and his colleagues found that adhering to LE8 guidelines to sustain a high CVH is related to increased life expectancy [12]. Furthermore, previous studies have revealed that LE8 scores were negatively associated with various chronic diseases among adults [13].

Given the tight relationship between COPD and CVD, promoting CVH through adhering to LE8 guidelines may be a feasible prevention and management strategy to alleviate the burden of COPD. Prior research has explored the correlation between LS7 and COPD [14], while the association between LE8 and COPD remains unclear to date. Hence, utilizing data from the National Health and Nutrition Examination Survey (NHANES), this study aims to investigate the associations between LE8 scores and COPD in a nationally representative U.S. population.

## Methods

### Study population

The NHANES is an ongoing cross-sectional survey conducted by the National Centers for Disease Control and Prevention, which utilized a multistage probability sampling design to investigate nutritional status, health behaviors, and physical examination results of the noninstitutionalized U.S. civilian population. The protocol for the NHANES study was granted by the ethics review board of the National Center for Health Statistics Research. Written informed consent was obtained from all participants. The more detailed information and data about NHANES are described at https://www.cdc.gov/nchs/nhanes/index.htm.

The six NHANES consecutive cycles (2007 to 2018) data were used in the presented study. Initially, 59,744 individuals from NHANES were included, and we began to exclude participants younger than 20 years of age (*N* = 24,974). Then, those who were missing or lacked demographic information and necessary covariates were excluded (*N* = 3,539) from this analysis. Participants who did not provide COPD assessment (*N* = 3), and without complete information for all 8 LE8 metrics (*N* = 11,484) were also eliminated. Finally, this analysis involved 19,774 participants from NHANES. A flow chart illustrating the selection of study participants is shown in Figure S1.

### Measurement of LE8

A detailed description of LE8 scoring algorithms for each of the metrics, based on the NAHNES data, can be found in Table S1. In short, each metric score of LE8 ranged from 0 to 100 points, and the overall LE8 score was calculated as the unweighted average of 8 metric scores. According to the AHA recommendations, individuals with a total LE8 score between 80 and 100, 79 and 50, and 0 and 49 were categorized as having high, moderate, or low cardiovascular health, respectively [10]. Diet metric was evaluated by the Healthy Eating Index (HEI)-2015 [15]. Studies regarding calculating HEI-2015 based on NHANES data have been previously published [16]. Table S2 summarizes the components and scoring standards of HEI-2015. In NHANES, self-report questionnaires were used to collect information about PA, nicotine exposure, and sleeping duration. The BP, height, and weight of participants were measured by professional staff during the physical examination. The BMI was calculated as the weight in kilograms divided by the height in meters squared. The blood samples were collected by NHANES researchers and sent to central laboratories for the determination of blood lipids, plasma glucose, and hemoglobin A1c.

### Ascertainment of COPD

In this NHANES cross-sectional study, COPD was determined as post-bronchodilator forced expiratory volume in the first second (FEV1)/forced vital capacity (FVC) < 0.7 for the 2007–2012 period or answer of “yes” to any of the following questions [17]: (1) “Has a doctor or other health professional ever told you that you have emphysema? (2) “Are you over 40 years old and have a history of smoking or chronic bronchitis, and take one of the following medications: mast cell stabilizers, inhaled corticosteroids, leukotriene modifiers, or selective phosphodiesterase-4 inhibitors?”.

### Assessment of covariates

Age was divided into three groups (20–39 years, 40–59 years, and ≥ 60 years). Race/ethnicity was classified into Non-Hispanic White, Non-Hispanic Black, Mexican American, and other races. Education levels have 5categories: less than 9th grade, 9th -11th grade (including 12th grade with no diploma), high school graduate (general educational development or equivalent), college graduate or above, and some college or associate’s degree. The poverty income ratio (PIR) was determined by dividing the monthly income of a family by the poverty thresholds. It was categorized into 3 levels: < 1.3, 1.3–3.5, and > 3.5. Three groups were classified according to their marital status as widowed/divorced/separated, never married, or married/living with their partner. In addition, lifestyle and health-related variables have been collected, as presented below: obesity status (kg/m² ≥ 30), alcohol consumption (never, former, and current), hypertension (an average systolic pressure ≥ 140 mmHg and diastolic pressure ≥ 90 mmHg in 3 consecutive tests), diabetes mellitus (DM) (fasting plasma glucose ≥ 126 mg/dL, 2-h plasma glucose ≥ 200 mg/dL, hemoglobin A1c ≥ 6.5%, or self-reported diabetes diagnosed by a professional doctor), CVD (the presence of a self-reported history of coronary heart disease, angina, myocardial infarction, or stroke by a trained health professional before the survey), chronic bronchitis (has a doctor or other health professional ever told you that you have chronic bronchitis) and asthma (has a doctor or other health professional ever told you that you have chronic asthma).

### Statistical analyses

According to the NHANES design, all statistical analyses in our study were weighted. The T-test analysis was used to compare continuous variables with their mean (standard error, SE). The Chi-square test was used to analyze counts and percentages of categorical variables. We used multivariate logistic regression models to estimate the adjusted odds ratio (AOR) and 95% confidence interval (CI) for the association of the LE8 score with COPD (low CVH levels as the reference). The crude model was without adjustment. Age, gender, obesity status, and race/ethnicity were adjusted in model 1, and model 2 was additionally adjusted for education level, marital status, PIR, and alcohol consumption. When the association of each LE8 metric score with COPD was evaluated, the remaining 7 LE8 components were further adjusted in model 2. A restricted cubic spline model was performed to explore the dose-response relationship between LE8 scores and COPD. Furthermore, subgroup analyses were conducted by different demographic characteristics such as age, gender, ethnicity/race, and so on. To assess the robustness of our results, we performed sensitivity analyses by adding other covariates (survey cycles, DM, hypertension, and CVD individually), and excluding underweight participants and those with asthma respectively. All statistical analyses were performed by R language (X64 Version 4.3.1, R Foundation for Statistical Computing). Statistical tests were 2-sided, and *P* < 0.05 was assumed to be statistical significance.

## Results

### Baseline characteristics

Baseline characteristics of the study population, by the presence of COPD, were summarized in Table 1. In this study, 19,774 participants were enrolled in six NHANES waves (2007–2018), representing the 145.2 million non-institutionalized U.S. population aged ≥ 20 years (Figure S1). The weighted average age (SE) of the participants was 47.77 (0.27) years old, of which 10,094 responders were females (51.5%). The average (SE) of LE8 score was 68.76 (0.26). There were 2,438 (9.8%), 13,222 (65.4%), and 4,084 (24.8%) participants with low (0–49), moderate (50–79), and high (80–100) CVH levels, respectively (Table S3). In the study population, the overall age-adjusted prevalence of COPD was 4.5% (0.20), with a higher prevalence observed among older, lower PIR participants and those with CVD (all the *P* value < 0.05). Participants without COPD exhibited significantly higher scores in PA, nicotine exposure, sleep health, blood lipids, blood glucose, and BP, compared with those with COPD (all the *P* value < 0.001). Table S3 shows the characteristics of the study population, by the CVH status as measured by LE8 scores.

**Table 1:** Characteristics of the study population, by the COPD, NHANES 2007–2018 (*n* = 19,774)

Characteristics	Age-adjusted prevalence of COPD^a^	Total	COPD		*P*-value^b^
			No	Yes	
**Participants**	4.5 (0.20)	19,744 (100.0)	18,743 (95.2)	1,001 (4.8)	**-**
**Age**,** years**	-	47.77 ± 0.27	47.14 ± 0.27	60.17 ± 0.47	**< 0.001**
20–39	0.7 (0.15)	6,507 (34.9)	6,455 (36.4)	52 (4.8)	**< 0.001**
40–59	5.2 (0.40)	6,651 (38.5)	6,312 (38.3)	339 (41.6)	
60-	9.7 (0.50)	6,586 (26.6)	5,976 (25.2)	610 (53.6)	
**Gender**	-				
Female	3.9 (0.28)	10,094 (51.5)	9,670 (51.7)	424 (47.1)	0.1
Male	5.0 (0.31)	9,650 (48.5)	9,073 (48.3)	577 (52.9)	
**Race/ethnicity**	-				
Non-Hispanic White	5.0 (0.27)	9,071 (70.9)	8,391 (70.2)	680 (84.9)	**< 0.001**
Non-Hispanic Black	3.2 (0.29)	3,933 (9.8)	3,780 (10.0)	153 (6.2)	
Mexican American	1.6 (0.28)	2,763 (7.5)	2,714 (7.8)	49 (1.6)	
Other races	3.6 (0.44)	3,977 (11.8)	3,858 (12.0)	119 (7.2)	
**Education level**	-				
Less than 9th grade	5.9 (0.83)	1,607 (4.0)	1,495 (3.9)	112 (6.7)	**< 0.001**
9-11th grade (includes 12th grade with no diploma)	6.9 (0.67)	2,563 (9.4)	2,378 (9.2)	185 (14.4)	
High school graduate/GED or equivalent	4.8 (0.41)	4,502 (22.6)	4,245 (22.5)	257 (25.4)	
Some college or AA degree	4.3 (0.32)	6,041 (31.8)	5,767 (31.9)	274 (29.3)	
College graduate or above	3.4 (0.30)	5,031 (32.1)	4,858 (32.5)	173 (24.2)	
**PIR**	-	3.09 ± 0.04	3.10 ± 0.04	2.90 ± 0.09	**< 0.05**
<1.3	6.7 (0.56)	5,936 (19.7)	5,570 (19.5)	366 (24.1)	**< 0.05**
1.3–3.5	4.5 (0.26)	7,464 (35.3)	7,101 (35.2)	363 (36.1)	
>3.5	3.7 (0.28)	6,344 (45.0)	6,072 (45.3)	272 (39.7)	
**Marital status**	-				
Never married	2.7 (0.43)	3,550 (17.5)	3,478 (18.1)	72 (5.3)	**< 0.001**
Widowed/Divorced/Separated	5.4 (0.50)	4,295 (17.9)	3,946 (17.3)	349 (28.8)	
Married/Living with partner	4.3 (0.24)	11,899 (64.6)	11,319 (64.6)	580 (65.9)	
**Alcohol consumption**	-				
Never	2.3 (0.41)	2,593 (10.1)	2,527 (10.3)	66 (5.4)	**< 0.001**
Former	6.6 (0.46)	3,127 (12.9)	2,838 (12.3)	289 (24.6)	
Current	4.3 (0.23)	14,024 (77.0)	13,378 (77.4)	646 (70.0)	
**Hypertension**	-				
No	3.8 (0.24)	11,349 (62.3)	10,982 (63.3)	367 (42.0)	**< 0.001**
Yes	5.0 (0.26)	8,395 (37.7)	7,761 (36.7)	634 (58.0)	
**DM**	-				
DM	5.5 (0.52)	3,283 (12.7)	3,008 (12.1)	275 (23.3)	**< 0.001**
IFG	6.0 (1.20)	954 (4.9)	891 (4.8)	63 (7.2)	
IGT	4.7 (0.90)	800 (3.6)	750 (3.5)	50 (4.9)	
No	4.1 (0.24)	14,707 (78.8)	14,094 (79.5)	613 (64.6)	
**Chronic bronchitis**	-				
No	3.4 (0.18)	18,579 (94.3)	17,880 (95.5)	699 (71.5)	**< 0.001**
Yes	18.9 (1.29)	1,165 (5.7)	863 (4.5)	302 (28.5)	
**Asthma**	-				
No	2.95 (0.18)	16,856 (85.3)	16,295 (86.7)	561 (57.2)	**< 0.001**
Yes	14.0 (0.82)	2,888 (14.7)	2,448 (13.3)	440 (42.8)	
**CVD**					
No	3.90 (0.21)	17,624 (91.6)	16,915 (92.5)	709 (75.2)	**< 0.001**
Yes	9.62 (1.05)	2,120 (8.4)	1,828 (7.5)	292 (24.8)	
**LE8 metric scores**					
Overall	-	68.76 ± 0.26	69.20 ± 0.27	60.02 ± 0.59	**< 0.001**
Diet	-	39.36 ± 0.55	39.43 ± 0.55	38.12 ± 1.27	0.3
Physical activity	-	74.35 ± 0.50	74.92 ± 0.52	63.11 ± 1.94	**< 0.001**
Nicotine exposure	-	72.21 ± 0.54	73.36 ± 0.54	49.67 ± 1.79	**< 0.001**
Sleep health	-	83.73 ± 0.31	83.99 ± 0.31	78.58 ± 1.24	**< 0.001**
Body mass index	-	60.10 ± 0.45	60.26 ± 0.47	57.05 ± 1.62	0.1
Blood lipids	-	64.72 ± 0.37	65.06 ± 0.37	58.02 ± 1.20	**< 0.001**
Blood glucose	-	85.74 ± 0.27	86.26 ± 0.28	75.51 ± 1.43	**< 0.001**
Blood pressure	-	69.86 ± 0.39	70.35 ± 0.40	60.13 ± 1.14	**< 0.001**

Footnotes: Continuous variables are presented as mean ± SE, and categorical variables are presented as n (weighted %)

^a^ Age-adjusted prevalence of COPD is presented as weight% (SE)

^b ^*P*-values were assessed by T-test (continuous variables) or by Chi-square test (categorical variables). *P*-values shown in bold were statistically significant.

Abbreviations: AA, Associate’s Degree; COPD, Chronic obstructive pulmonary disease; CVD, Cardiovascular disease; DM, Diabetes mellitus; GED, General educational development; IFG, Impaired fasting glycaemia; IGT, Impaired glucose tolerance; LE8, Life’s Essential 8; NHANES, National Health and Nutrition Examination Survey; PIR, Poverty income ratio; SE, Standard error

### LE8 score and COPD

**Fig. 1:**
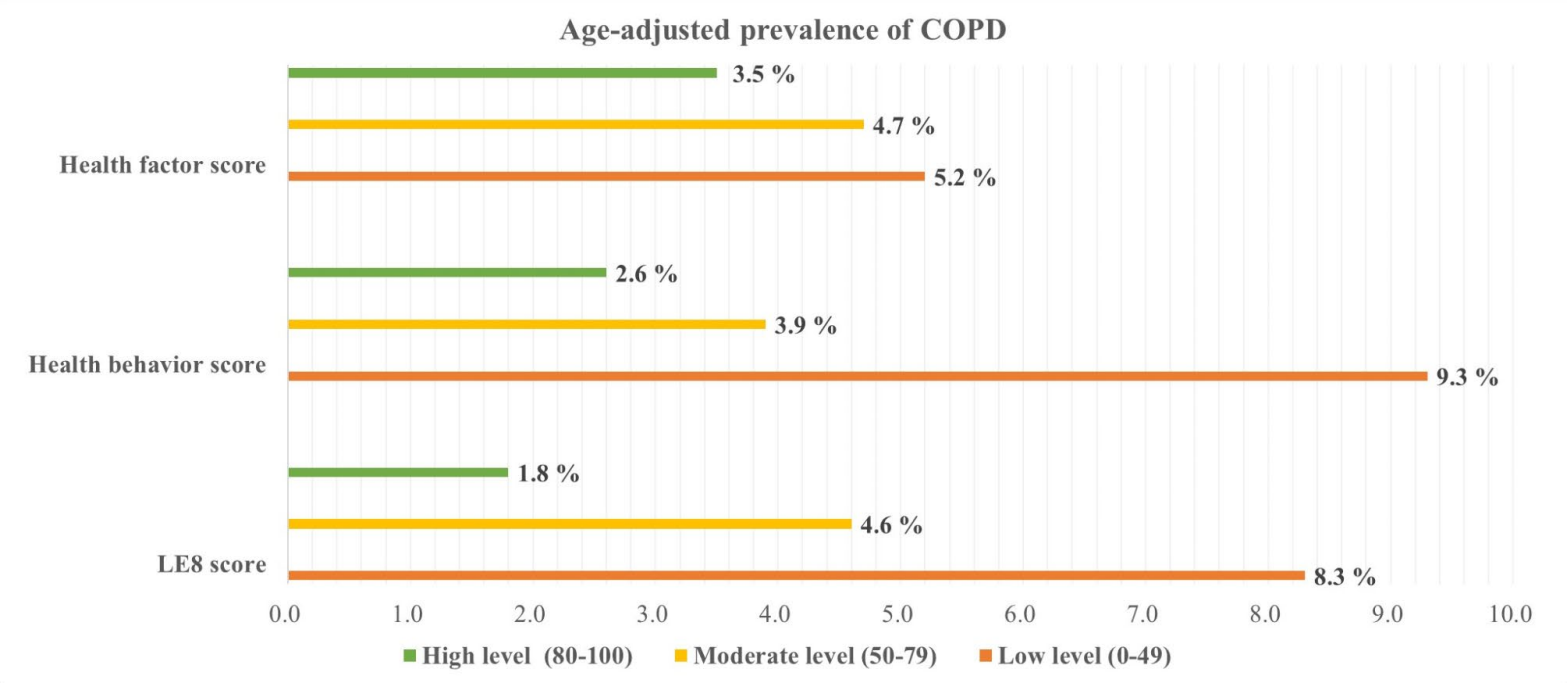
Age-adjusted prevalence of COPD in different levels of the LE8 score, health behavior score, and health factor score, NHANES 2007–2018 (*n* = 19,774)

**Fig. 2:**
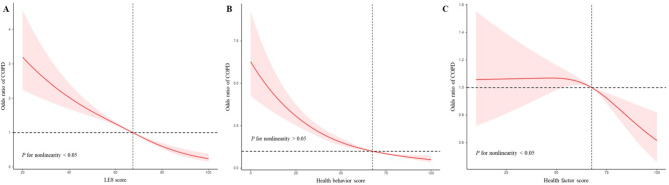
Dose-response relationships between the LE8 score (**A**), health behavior score (**B**), health factor score (**C**), and COPD, NHANES 2007–2018 (*n* = 19,774)

A lower age-adjusted prevalence of COPD was observed among participants with a higher LE8 score (Fig. 1). After adjustment for potential covariates, the lower AOR of COPD was significantly associated with a moderate CVH level (AOR = 0.477, 95% CI: 0.394, 0.578) and a high CVH level (AOR = 0.169, 95% CI: 0.115, 0.249), compared to the low CVH group (Table 2). The total LE8 score and the odds ratio (OR) of COPD exhibited an inverse nonlinear dose-response relationship (*P* for nonlinearity < 0.05) (Fig. 2A). Moreover, Similar trends were observed for participants with higher LE8 component scores of sleep health (AOR = 0.670, 95% CI: 0.525, 0.855), and nicotine exposure (AOR = 0.164, 95% CI: 0.123, 0.218) (Table 3).

**Table 2:** Associations of the LE8 score, health behavior score, and health factor score with COPD, NHANES 2007–2018 (*n* = 19,774)

	Crude model			Model 1			Model 2	
	COR (95% CI)	*P*-value		AOR (95% CI)	*P*-value		AOR (95% CI)	*P*-value
**LE8 score**								
Low CVH (0–49)	Reference	-		Reference	-		Reference	-
Moderate CVH (50–79)	**0.416 (0.351**,** 0.494)**	**< 0.001**		**0.416 (0.345**,** 0.502)**	**< 0.001**		**0.477 (0.394**,** 0.578)**	**< 0.001**
High CVH (80–100)	**0.109 (0.075**,** 0.157)**	**< 0.001**		**0.130 (0.089**,** 0.192)**	**< 0.001**		**0.169 (0.115**,** 0.249)**	**< 0.001**
*P* for trend		**< 0.001**			**< 0.001**			**< 0.001**
**Health behavior score**								
Low (0–49)	Reference	-		Reference	-		Reference	-
Moderate (50–79)	**0.387 (0.318**,** 0.470)**	**< 0.001**		**0.367 (0.297**,** 0.454)**	**< 0.001**		**0.419 (0.337**,** 0.521)**	**< 0.001**
High (80–100)	**0.271 (0.210**,** 0.349)**	**< 0.001**		**0.244 (0.186**,** 0.320)**	**< 0.001**		**0.300 (0.223**,** 0.404)**	**< 0.001**
*P* for trend		**< 0.001**			**< 0.001**			**< 0.001**
**Health factor score**								
Low (0–49)	Reference	-		Reference	-		Reference	-
Moderate (50–79)	**0.711 (0.556**,** 0.908)**	**< 0.05**		0.796 (0.609, 1.039)	0.093		0.850 (0.645, 1.120)	0.245
High (80–100)	**0.304 (0.228**,** 0.405)**	**< 0.001**		**0.533 (0.381**,** 0.745)**	**< 0.001**		**0.603 (0.426**,** 0.852)**	**< 0.05**
*P* for trend		**< 0.001**			**< 0.001**			**< 0.05**

Footnotes: The crude model was unadjusted. Model 1 was adjusted for age, gender, race/ethnicity, and obesity status. Model 2 was additionally adjusted for education level, marital status, PIR, and alcohol consumption. The results of COR (95% CI), AOR (95% CI), and *P*-value shown in bold were statistically significant. *P*-value < 0.05 or *P*-value < 0.001

Abbreviations: AOR, Adjusted odds ratio; CI, Confidence interval; COPD, Chronic obstructive pulmonary disease; COR, Crude odds ratio; CVH, Cardiovascular health; LE8, Life’s Essential 8; NHANES, National Health and Nutrition Examination Survey; PIR, Poverty income ratio

**Table 3:** Associations of LE8 components with COPD, NHANES 2007–2018 (*n* = 19,774)

LE8 components	Crude model			Model 1			Model 2	
	COR (95% CI)	*P*-value		AOR (95% CI)	*P*-value		AOR (95% CI)	*P*-value
**Diet**								
Low (0–49)	Reference	-		Reference	-		Reference	-
Moderate (50–79)	0.950 (0.797, 1.133)	0.563		0.793 (0.663, 0.949)	0.012		0.965 (0.790, 1.178)	0.720
High (80–100)	0.869 (0.704, 1.073)	0.190		**0.636 (0.518**,** 0.782)**	**< 0.001**		0.958 (0.757, 1.212)	0.715
*P* for trend		0.178			**< 0.001**			0.679
**Physical activity**								
Low (0–49)	Reference	-		Reference	-		Reference	-
Moderate (50–79)	**0.564 (0.363**,** 0.877)**	**< 0.05**		0.655 (0.423, 1.015)	0.058		0.824 (0.521, 1.306)	0.405
High (80–100)	**0.568 (0.472**,** 0.684)**	**< 0.001**		**0.750 (0.615**,** 0.914)**	**< 0.05**		0.926 (0.747, 1.149)	0.480
*P* for trend		**< 0.001**			**< 0.05**			0.510
**Nicotine exposure**								
Low (0–49)	Reference	-		Reference	-		Reference	-
Moderate (50–79)	1.010 (0.836, 1.222)	0.914		**0.536 (0.430**,** 0.669)**	**< 0.001**		**0.587 (0.464**,** 0.743)**	**< 0.001**
High (80–100)	**0.168 (0.132**,** 0.214)**	**< 0.001**		**0.132 (0.103**,** 0.169)**	**< 0.001**		**0.164 (0.123**,** 0.218)**	**< 0.001**
*P* for trend		**< 0.001**			**< 0.001**			**< 0.001**
**Sleep health**								
Low (0–49)	Reference	-		Reference	-		Reference	-
Moderate (50–79)	**0.682 (0.542**,** 0.858)**	**< 0.05**		**0.699 (0.548**,** 0.890)**	**< 0.05**		0.789 (0.600, 1.038)	0.090
High (80–100)	**0.585 (0.472**,** 0.726)**	**< 0.001**		**0.530 (0.422**,** 0.664)**	**< 0.001**		**0.670 (0.525**,** 0.855)**	**< 0.05**
*P* for trend		**< 0.001**			**< 0.001**			**< 0.05**
**Body mass index**								
Low (0–49)	Reference	-		Reference	-		Reference	-
Moderate (50–79)	0.973 (0.768, 1.231)	0.815		0.890 (0.695, 1.139)	0.349		0.953 (0.741, 1.228)	0.707
High (80–100)	0.792 (0.613, 1.023)	0.074		0.867 (0.667, 1.128)	0.285		0.935 (0.713, 1.227)	0.625
P for trend		0.085			0.263			0.614
**Blood lipids**								
Low (0–49)	Reference	-		Reference	-		Reference	-
Moderate (50–79)	**0.545 (0.423**,** 0.703)**	**< 0.001**		0.705 (0.541, 0.918)	0.010		0.767 (0.576, 1.023)	0.070
High (80–100)	**0.730 (0.621**,** 0.858)**	**< 0.001**		**0.821 (0.694**,** 0.971)**	**< 0.05**		0.821 (0.668, 1.009)	0.060
*P* for trend		**< 0.001**			**< 0.05**			0.061
**Blood glucose**								
Low (0–49)	Reference	-		Reference	-		Reference	-
Moderate (50–79)	0.823 (0.642, 1.055)	0.122		0.894 (0.688, 1.162)	0.400		0.925 (0.675, 1.268)	0.624
High (80–100)	**0.362 (0.269**,** 0.488)**	**< 0.001**		**0.613 (0.442**,** 0.851)**	**< 0.05**		0.701 (0.479, 1.027)	0.067
*P* for trend		**< 0.001**			**0.001**			**< 0.05**
**Blood pressure**								
Low (0–49)	Reference	-		Reference	-		Reference	-
Moderate (50–79)	0.750 (0.591, 0.953)	0.019		1.144 (0.890, 1.471)	0.289		1.209 (0.931, 1.570)	0.152
High (80–100)	**0.565 (0.448**,** 0.713)**	**< 0.001**		1.159 (0.896, 1.499)	0.257		1.228 (0.929, 1.623)	0.147
*P* for trend		**< 0.001**			0.302			0.187

Footnotes: The crude model was unadjusted. Model 1 was adjusted for age, gender, and race/ethnicity. Model 2 was additionally adjusted for education level, marital status, PIR, alcohol consumption and the remaining 7 LE8 components. The results of COR (95% CI), AOR (95% CI), and *P*-value shown in bold were statistically significant. *P*-value < 0.05 or *P*-value < 0.001

Abbreviations: AOR, Adjusted odds ratio; CI, Confidence interval; COPD, Chronic obstructive pulmonary disease; COR, Crude odds ratio; LE8, Life’s Essential 8; NHANES, National Health and Nutrition Examination Survey; PIR, Poverty income ratio

### Health behavior score and COPD

A lower age-adjusted prevalence of COPD was observed among participants with a higher health behavior score (Fig. 1). After adjustment for potential covariates, the AOR of COPD was significantly lower in participants with a moderate health behavior score (AOR = 0.419, 95% CI: 0.337, 0.521) and a high health behavior score (AOR = 0.300, 95% CI: 0.223, 0.404), when compared to those with a low health behavior score (Table 2). Moreover, an inverse linear dose-response relationship between health behavior scores and COPD was observed (Fig. 2B).

### Health factor score and COPD

A lower age-adjusted prevalence of COPD was observed among participants with a higher health factor score (Fig. 1). After adjustment for potential covariates, the AOR of COPD was significantly lower in participants with a moderate health factor score (AOR = 0.419, 95% CI: 0.337, 0.521) and a high health factor score (AOR = 0.300, 95% CI: 0.223, 0.404), when compared to those with a low health factor score (Table 2). Moreover, an inverse nonlinear dose-response relationship between health factor scores and COPD was observed (Fig. 2C).

### Subgroup and sensitivity analyses

The results of subgroup analyses are presented in Table 4. Compared with participants with low CVH status, there was a significantly lower AOR of COPD in participants who attained moderate and high CVH levels in all subgroups, except individuals aged 20–39 years. A significant interaction was found between LE8 and age, as well as marital status with COPD (*P*-interaion < 0.05). Middle-aged and male participants showed a stronger reverse association between LE8 scores and COPD.

**Table 4:** Subgroup analysis of the association of LE8 score with COPD, NHANES 2007–2018 (*n* = 19,774)

Subgroups	LE8 score							*P*-interaction
	Low CVH (0–49)		Moderate CVH (50–79)			High CVH (80–100)		
			AOR (95% CI)	*P*-value		AOR (95% CI)	*P*-value	
**Gender**								
Female	Reference		**0.610 (0.468**,** 0.793)**	**< 0.001**		**0.273 (0.161**,** 0.461)**	**< 0.001**	0.171
Male	Reference		**0.463 (0.355**,** 0.605)**	**< 0.001**		**0.142 (0.083**,** 0.242)**	**< 0.001**	
**Age**,** years**								
20–39	Reference		1.527 (0.502, 4.646)	0.451		0.713 (0.161, 3.153)	0.652	**< 0.05**
40–59	Reference		**0.613 (0.460**,** 0.816)**	**< 0.05**		**0.133 (0.072**,** 0.247)**	**< 0.001**	
60-	Reference		**0.535 (0.409**,** 0.699)**	**< 0.001**		**0.342 (0.211**,** 0.556)**	**< 0.001**	
**Race/ethnicity**								
Non-Hispanic White	Reference		**0.523 (0.423**,** 0.647)**	**< 0.001**		**0.216 (0.144**,** 0.323)**	**< 0.001**	0.107
Non-Hispanic Black	Reference		**0.496 (0.336**,** 0.731)**	**< 0.001**		**0.243 (0.099**,** 0.596)**	**< 0.05**	
Mexican American	Reference		**0.507 (0.274**,** 0.938)**	**< 0.05**		**0.098 (0.010**,** 0.948)**	**< 0.05**	
Other races	Reference		**0.553 (0.335**,** 0.912)**	**< 0.05**		**0.071 (0.025**,** 0.198)**	**< 0.001**	
**Education level**								
Less than 9th grade	Reference		**0.540 (0.295**,** 0.987)**	**< 0.05**		**0.058 (0.015**,** 0.227)**	**< 0.001**	0.732
9-11th grade (including 12th grade with no diploma)	Reference		**0.513 (0.309**,** 0.851)**	**< 0.05**		**0.117 (0.035**,** 0.395)**	**< 0.001**	
High school graduate/GED or equivalent	Reference		**0.612 (0.436**,** 0.859)**	**< 0.05**		**0.220 (0.085**,** 0.570)**	**< 0.05**	
Some college or AA degree	Reference		**0.520 (0.358**,** 0.754)**	**< 0.001**		**0.217 (0.102**,** 0.461)**	**< 0.001**	
College graduate or above	Reference		**0.350 (0.190**,** 0.643)**	**< 0.001**		**0.153 (0.074**,** 0.316)**	**< 0.001**	
**Marital status**								
Never married	Reference		0.618 (0.330, 1.157)	0.131		**0.115 (0.028**,** 0.476)**	**< 0.05**	**< 0.05**
Widowed/Divorced/Separated	Reference		**0.449 (0.326**,** 0.618)**	**< 0.001**		**0.075 (0.037**,** 0.153)**	**< 0.001**	
Married/Living with partner	Reference		**0.558 (0.437**,** 0.713)**	**< 0.001**		**0.261 (0.170**,** 0.400)**	**< 0.001**	
**PIR**								
< 1.3	Reference		**0.413 (0.306**,** 0.557)**	**< 0.001**		**0.116 (0.039**,** 0.349)**	**< 0.001**	0.437
1.3–3.5	Reference		**0.531 (0.370**,** 0.761)**	**< 0.001**		**0.240 (0.123**,** 0.469)**	**< 0.001**	
> 3.5	Reference		0.704 (0.458, 1.081)	0.107		**0.269 (0.144**,** 0.500)**	**< 0.001**	
**Alcohol consumption**								
Never	Reference		0.603 (0.323, 1.124)	0.110		**0.162 (0.048**,** 0.546)**	**< 0.05**	0.647
Former	Reference		**0.445 (0.296**,** 0.667)**	**< 0.001**		**0.120 (0.039**,** 0.367)**	**< 0.001**	
Current	Reference		**0.559 (0.440**,** 0.711)**	**< 0.001**		**0.231 (0.155**,** 0.342)**	**< 0.001**	

Footnotes: The multivariable logistic regression model was adjusted for age, gender, race/ethnicity, education level, marital status, PIR, and alcohol consumption. The results of AOR (95% CI), *P*-interaction, and *P*-value shown in bold were statistically significant. *P*-value < 0.05 or *P*-value < 0.001

Abbreviations: AA, Associate’s Degree; AOR, Adjusted odds ratio; CI, Confidence interval; COPD, Chronic obstructive pulmonary disease; CVH, Cardiovascular health; GED, General educational development; LE8, Life’s Essential 8; NHANES, National Health and Nutrition Examination Survey; PIR, Poverty income ratio

The sensitivity analysis results were in line with our primary findings. After additional adjustments for the survey cycle, DM, hypertension, and CVD respectively, the moderate and high CVH groups remained significantly associated with a lower AOR of COPD, compared with the low CVH group (Table S4). In addition, the association between LE8 and COPD remained robust, even after excluding underweight individuals and those with asthma, respectively (Table S5, Table S6).

## Discussion

In this nationally representative cross-sectional study of the U.S. population, we found significant inverse associations between LE8 and its metric scores (nicotine exposure and sleep health) and the prevalence of COPD. Furthermore, health behavior scores and health factor scores were associated with COPD in similar trends. Our primary results remained robust even when other potential covariates were taken into account and when participants who were underweight and had asthma were excluded in sensitivity analyses. In addition, subgroup analyses showed that the inverse associations between LE8 scores and COPD were stronger among middle-aged and male populations.

CVD is one of the most severe and common comorbidities of COPD, with each condition complicating the prognosis of the other [18, 19]. Previous studies primarily investigated single factors for COPD, while the presented study using comprehensive LE8 measurement suggested that individuals with higher LE8 scores (higher CVH levels) showed a lower prevalence of COPD, which is consistent with previous relevant studies. A cross-sectional study including 6,352 U.S. adults indicated that LS7 was negatively linked to both lung function and the odds of COPD, and a positive linear relationship was also identified between the LS7 score and lung function [20]. Given that LS7 has the same metrics as LE8 (except for sleep health), despite the limitations of LS7, this finding somewhat supports our main results. Chen and his colleagues found that the prevalence of CVD was significantly higher in patients with COPD than in patients without COPD [21]. A meta-analysis also showed that patients with COPD have a significantly higher likelihood of being diagnosed with CVD (OR = 2.46; 95% CI: 2.02-3.00; *P* < 0.0001) in comparison to the non-COPD population [22]. These findings underscored the need to raise awareness regarding the coexistence of COPD and CVD. It also revealed the potential benefits of improving the CVH through lifestyle changes to relieve systemic and not systematic inflammation (especially smoking cessation) in patients with CVD to reduce risks of COPD [19, 21, 22]. In addition, there is evidence that FEV1, the hallmark of disease progression in COPD, is likely associated with higher risks of DM, hypertension, and asthma [23]. After controlling for these confounding variables (DM, hypertension, and CVD) and excluding participants with asthma, our primary results remained robust.

The association of LE8 with COPD is multifactorial and not fully understood to date. Based on the present evidence, this may be largely explained by the LE8 sharing the common risk factors for COPD [7]. Specifically, first, diet patterns (DASH, Mediterranean, and Balanced Diets) may influence inflammation, oxidative stress, antioxidant depletion, mucus hypersecretion, alveolar wall destruction, defective tissue repair, and airway remodeling, all of which are part of the pathogenesis of COPD [24]. Second, recent studies have established that exercise can promote the differentiation of lung tissue stem cell and reshape blood vessel formation, thereby enhancing lung ventilation function [25, 26]. This finding unveiled the potential beneficial role of PA on COPD. Third, smoking is widely recognized as the primary cause of COPD, and our results also confirmed it. Tobacco smoke exposure would lead to changes in the innate immune system, resulting in significant and chronic inflammation of the lung. This in turn triggers other pathological changes, such as remodeling and destruction of lung tissue [27–29]. Fourth, sleep disturbance is among the most frequently reported symptoms by COPD patients. It has been reported that sleep deprivation is associated with mild decreases in FVC (-5%) and FEV1 (-6%), and patients with severe COPD tended to have poor sleep quality (oxygen desaturation) relative to normative populations [30–32]. Moreover, it is notable that metabolic syndrome (MetS) is prevalent in patients with COPD [33], and its components constitute the health factors in LE8 [34]. A review study by Baffi et al. showed that the individual MetS components, including dyslipidemia, fasting hyperglycemia, abdominal obesity, and hypertension, were independently associated with lung function impairment [35]. Another research further revealed that MetS and increased adiposity increase the risks of CVD in COPD patients, and the underlying mechanisms mainly include vascular oxidative stress and inflammation, partly facilitated by pro-inflammatory adipokines secreted by adipose tissue [36, 37]. This may explain the relationship between LE8 and COPD.

Additionally, our study unveiled a non-linear relationship between the LE8 score and health factor score with the OR of COPD, as well as a linear association between the health behavior score and COPD. The OR for the LE8 score and health behavior score associated with COPD decreased significantly in the lower range of the respective scores and subsequently remained stable at higher values. Conversely, the correlation between COPD and the health factor score exhibited an opposite trend, with OR remaining stable when the health factor score was low and decreasing sharply as the score increased. Furthermore, we employed multivariate logistic regression models to examine the relationships between all 8 LE8 components and COPD. The results suggested that: (1) apart from nicotine exposure (*P*-value < 0.001) and sleep health metrics (*P*-value < 0.05) in LE8, the effects of the other LE8 components on COPD were relatively limited (*P*-value > 0.05); (2) the OR of the nicotine exposure metrics (0.164) was comparable to that of the full LE8 (0.169). From this finding, the negative association of LE8 with COPD may be mainly contributed by cigarette smoking; (3) the inverse correlation between sleep health metrics in LE8 and COPD was supported by related studies that showed the prevalence of poor sleep quality is significantly associated with severe COPD [38]. This finding demonstrated adding a sleep health metric for LE8 to assess CVH is crucial and increases the sensitivity of LE8.

To the best of our knowledge, this is the first study to examine the relationship between LE8 and COPD in a large nationally representative sample of U.S. adults. Additionally, we investigated the dose-response relationship between LE8 scores and COPD. To date, these findings have not been reported in prior studies. Our results unveiled the significance of sustaining a higher CVH level for preventing COPD. The primary implication of this study is as follows: adhering to LE8 guidelines to sustain a higher CVH level, especially smoking cessation, maybe a plausible prevention approach for COPD, which provides important insights for caregivers and clinical staff. However, to better establish this, future studies are required to conduct multifactorial interventions based on key LE8 components to examine its impacts on COPD risks. There are several limitations to note in our study. (1) The evaluation of health behaviors in LE8 (including diet, nicotine exposure, PA, and sleep health) relied on self-reported questionnaires, which were more likely to cause recall bias. Also, it is to be noted that there are some limitations to the ascertainment of COPD in this study, including reliance on spirometry or self-reporting rather than doctor diagnosis, not considering chronic respiratory symptoms), the absence of exclusion of asthma patients (the 43% of the COPD subjects reported asthma). These shortcomings in the diagnostic criteria may lead to biased estimates of COPD prevalence, potentially affecting the accuracy and validity of our results. (2) A large number of participants (*n* = 15,026) from the NHANES dataset were excluded due to missing data, and we were unable to establish whether the missing data occurred completely at random, which may potentially create a major selection bias and impact on the reliability and validity of study results. (3) Evidence suggests a significant association between underweight status and both COPD and lower lung function [39, 40]. However, the LE8 algorithm considers individuals with BMI < 25 kg/m^2^ as optimal (100 points) and does not take into account the underweight population (BMI < 18.5 kg/m^2^). Thus, this methodological limitation may mislead our results. (4) Despite adjusting for several potential confounders, our analysis may be affected by unmeasured or insufficiently measured confounders. (5) Since the presented study was a cross-sectional design, we are unable to determine the causality and temporality of LE8 and COPD. Future prospective studies are warranted to investigate the relationship further.

## Conclusions

In summary, the LE8 score, health behavior score, and health factor score were inversely associated with COPD in the general U.S. population. In addition, a nonlinear dose-response association was observed between COPD and both the LE8 score and health factor score, while the health behavior score exhibited a linear dose-response relationship with COPD. This cross-sectional study shows an inverse association between LE8 and spirometric/self-reported COPD, primarily driven by nicotine exposure metric, as confirmed by analyses of LE8 components.

## Electronic supplementary material

Below is the link to the electronic supplementary material.

**Supplementary Material 1: Figure S1. **Flowchart of selection of participants. **Table S1** Definition and scoring approach for the Life’s Essential 8. **Table S2** Healthy Eating Index-2015 components of point values, and scoring standards. **Table S3** Characteristics of the study population, by the LE8-evaluated CVH levels, NHANES 2007–2018 (*n* = 19,774). **Table S4** Association of LE8 scores with COPD for additional adjustments, NHANES 2007–2018 (*n* = 19,744). **Table S5** Association of the LE8 score with COPD, excluding 277 underweight participants. **Table S6** Association of the LE8 score with COPD, excluding 2,888 participants with asthma.

## Data Availability

The datasets generated and analyzed during the present study are available from the NHANES databases (Available from https://www.cdc.gov/nchs/nhanes/participant.htm).

## Abbreviations

AHA: American Heart Association
AOR: Adjusted odds ratio
BMI: Body mass index
BP: Blood pressure
COPD: Chronic obstructive pulmonary disease
CVD: Cardiovascular disease
CVH: Cardiovascular health
DM: Diabetes mellitus
OR: Odds ratio
LE8: Life’s Essential 8
LS7: Life’s Simple 7
MetS: Metabolic syndrome
HEI: Healthy eating index
NHANES: National Health and Nutrition Examination Surveys
PA: Physical activity
PIR: Poverty income ratio
