# Supplementary material for: Association of life’s essential 8 with chronic obstructive pulmonary disease: a population-based analysis of NHANES 2007–2018
Source: BMC Public Health. 2024 Nov 13;24:3144. doi: 10.1186/s12889-024-20534-5 (PMC11558873; doi:10.1186/s12889-024-20534-5)
Supplement: Supplementary file 1 — Supplementary Material 1: Figure S1. Flowchart of selection of participants. Table S1 Definition and scoring approach for the Life’s Essential 8. Table S2 Healthy Eating Index-2015 components of point values, and scoring standards. Table S3 Characteristics of the study population, by the LE8-evaluated CVH levels, NHANES 2007–2018 (n = 19,774). Table S4 Association of LE8 scores with COPD for additional adjustments, NHANES 2007–2018 (n = 19,744). Table S5 Association of the LE8 score with COPD, excluding 277 underweight participants. Table S6 Association of the LE8 score with COPD, excluding 2,888 participants with asthma. [file 12889_2024_20534_MOESM1_ESM.docx]

**Supplementary Materials**

**Association of Life's Essential 8 with Chronic Obstructive Pulmonary Disease: A Population-based Analysis of NHANES 2007-2018**

**Table of contents**

[**Figure S1 Flowchart of selection of participants.** 3](#_Toc176875385)

[**Table S1** **Definition and scoring approach for the American Heart Association’s Life’s Essential 8.** 4](#_Toc176875386)

[**Table S2 Healthy Eating Index-2015 components, point values, and standards for scoring.** 6](#_Toc176875387)

[**Table S3 Characteristics of the study population, by the LE8-evaluated CVH levels, NHANES 2007-2018 (n = 19,774).** 7](#_Toc176875388)

[**Table S4 Association of LE8 scores with COPD for additional adjustments, NHANES 2007-2018 (n = 19,744).** 9](#_Toc176875389)

[**Table S5 Association of the LE8 score with COPD, excluding 277 underweight participants.** 10](#_Toc176875390)

[**Table S6 Association of the LE8 score with COPD, excluding 2.888 patients with asthma.** 11](#_Toc176875391)

**
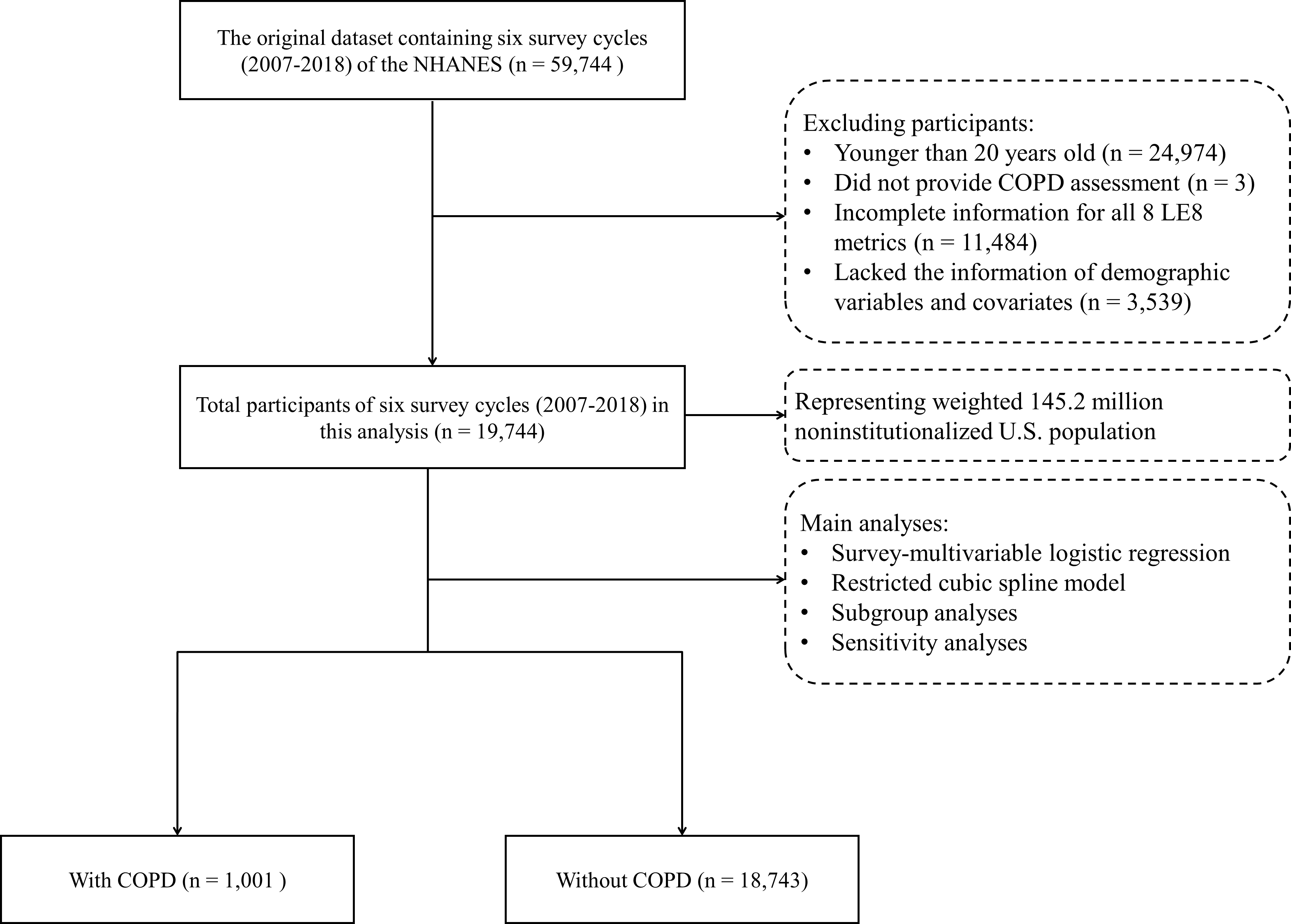
**

**Figure S1 Flowchart of selection of participants.**

Abbreviations: COPD, Chronic obstructive pulmonary disease; LE8, Life’s Essential 8; NHANES, National Health and Nutrition Examination Survey.

| **Table S1** **Definition and scoring approach for the American Heart Association’s Life’s Essential 8.** | | | |
| --- | --- | --- | --- |
| **Domain** | **CVH metric** | **Method of measurement** | **Quantification and scoring of CVH metric: adults (≥ 20 years of age)** |
| **Health behaviors** | Diet | Healthy Eating Index-2015 diet score percentile | Quantiles of DASH-style diet adherence HEI-2015 (population)  Scoring (Population):  Points Quantile  100 ≥ 95th percentile (top/ideal diet)  80 75th-94th percentile  50 50th-74th percentile  25 25th-49th percentile  0 1st-24th percentile (bottom/least ideal quartile) |
|  | Physical activity | Self-reported minutes of moderate or vigorous physical activity per week | Metric: Minutes of moderate (or greater) intensity activity per week  Scoring:  Points Minutes  100 ≥ 150  90 120-149  80 90-119  60 60-89  40 30-59  20 1-29  0 0 |
|  | Nicotine exposure | Self-reported use of cigarettes or inhaled NDS | Metric: Combustible tobacco use and/or inhaled NDS use; or secondhand smoke exposure  Scoring:  Points Status  100 Never smoker  75 Former smoker, quit ≥5 years  50 Former smoker, quit 1-< 5 years  25 Former smoker, quit < 1 year, or currently using inhaled NDS  0 Current smoker  Subtract 20 points (unless score is 0) for living with active indoor smoker in home |
|  | Sleep health | Self-reported average hours of sleep per night | Metric: Average hours of sleep per night  Scoring:  Points Level  100 7-< 9  90 9-< 10  70 6-< 7  40 5-< 6 or ≥ 10  20 4-< 5  0 < 4 |
| **Health factors** | Body mass index | Body weight (kg) divided by height squared (m^2^) | Metric: Body mass index (kg/m^2^)  Scoring:  Points Level  100 < 25  70 25.0-29.9  30 30.0-34.9  15 35.0-39.9  0 ≥ 40.0 |
|  | Blood lipids | Plasma total and HDL-cholesterol with calculation of non-HDL-cholesterol | Metric: Non-HDL-cholesterol (mg/dL)  Scoring:  Points Level  100 < 130  60 130-159  40 160-189  20 190-219  0 ≥ 220  If drug-treated level, subtract 20 points |
|  | Blood glucose | Fasting blood glucose or casual hemoglobin A1c | Metric: Fasting blood glucose (mg/dL) or hemoglobin A1c (%)  Scoring:  Points Level  100 No history of diabetes and FBG < 100 (or HbA1c < 5.7)  60 No diabetes and FBG 100 – 125 (or HbA1c 5.7-6.4) (Pre-diabetes)  40 Diabetes with HbA1c < 7.0  30 Diabetes with HbA1c 7.0-7.9  20 Diabetes with HbA1c 8.0-8.9  10 Diabetes with Hb A1c 9.0-9.9  0 Diabetes with HbA1c ≥ 10.0 |
|  | Blood pressure | Appropriately measured systolic and diastolic blood pressure | Metric: Systolic and diastolic blood pressure (mm Hg)  Scoring:  Points Level  100 < 120/< 80 (Optimal)  75 120-129/< 80 (Elevated)  50 130-139 or 80-89 (Stage 1 hypertension)  25 140-159 or 90-99  0 ≥ 160 or ≥ 100  Subtract 20 points if treated level |
| References:  [1] Lloyd-Jones DM, Hong Y, Labarthe D, et al. Defining and setting national goals for cardiovascular health promotion and disease reduction: the American Heart Association's strategic Impact Goal through 2020 and beyond. Circulation. 2010;121(4):586-613. doi:10.1161/CIRCULATIONAHA.109.192703  [2] Lloyd-Jones DM, Allen NB, Anderson CAM, et al. Life's Essential 8: Updating and Enhancing the American Heart Association's Construct of Cardiovascular Health: A Presidential Advisory From the American Heart Association. Circulation. 2022;146(5):e18-e43. doi:10.1161/CIR.0000000000001078  Abbreviations: CVH, Cardiovascular health; FBG, Fasting blood glucose; HbA1c: Hemoglobin A1c; HDL, High-density lipoprotein; NDS, Nicotine-delivery system; HEI, Healthy Eating Index. | | | |

| **Table S2 Healthy Eating Index-2015 components, point values, and standards for scoring.** | | | |
| --- | --- | --- | --- |
| **Component** | **Maximum points** | **Standard for maximum score** | **Standard for minimum score of zero** |
| **Adequacy** |  |  |  |
| Total Fruits^a^ | 5 | ≥0.8 c equivalents/1,000 kcal | No fruit |
| Whole Fruits | 5 | ≥0.4 c equivalents/1,000 kcal | No whole fruit |
| Total Vegetables | 5 | ≥1.1 c equivalents/1,000 kcal | No vegetables |
| Greens and Beans | 5 | ≥0.2 c equivalents/1,000 kcal | No dark green vegetables or beans and peas |
| Whole Grains | 10 | ≥1.5 oz equivalents/1,000 kcal | No whole grains |
| Dairy | 10 | ≥1.3 c equivalents/1,000 kcal | No dairy |
| Total Protein Foods | 5 | ≥2.5 oz equivalents/1,000 kcal | No protein foods |
| Seafood and Plant Proteins | 5 | ≥0.8 c equivalents/1,000 kcal | No seafood or plant proteins |
| Fatty Acids | 10 | (PUFAs+MUFAs)/SFAs≥2.5 | (PUFAs+MUFAs)/SFAs ≤1.2 |
| **Moderation** |  |  |  |
| Refined Grains | 10 | ≤1.8 oz equivalents/1,000 kcal | ≥4.3 oz equivalents/1,000 kcal |
| Sodium | 10 | ≤1.1 g/1,000 kcal | ≥2.0 g/1,000 kcal |
| Added Sugars | 10 | ≤6.5% of energy | ≥26% of energy |
| Saturated Fats | 10 | ≤8% of energy | ≥16% of energy |
| Reference:  [1] Krebs-Smith SM, Pannucci TE, Subar AF, et al. Update of the Healthy Eating Index: HEI-2015 [published correction appears in J Acad Nutr Diet. 2019 Aug 20;:]. J Acad Nutr Diet. 2018;118(9):1591-1602. doi:10.1016/j.jand.2018.05.021  Abbreviations: MUFAs, Monounsaturated fatty acids; PUFAs, Polyunsaturated fatty acids; SFAs, saturated fatty acids. | | | |

| **Table S3 Characteristics of the study population, by the LE8-evaluated CVH levels, NHANES 2007-2018 (n = 19,774).** | | | | | |
| --- | --- | --- | --- | --- | --- |
| **Characteristics** | **Total** | **LE8 score** | | | ***P*-value^a^** |
|  |  | **Low CVH (0-49)** | **Moderate CVH (50-79)** | **High CVH (80-100)** |  |
| **Participants** | 19,744 (100.0) | 2,438 (9.8) | 13,222 (65.4) | 4,084 (24.8) | - |
| **Age, years** | 47.77 ± 0.27 | 54.27 ± 0.33 | 49.06 ± 0.29 | 41.80 ± 0.43 | **< 0.001** |
| 20-39 | 6,507 (34.9) | 366 (17.8) | 3,977 (31.7) | 2,164 (50.2) | **< 0.001** |
| 40-59 | 6,651 (38.5) | 936 (43.5) | 4,512 (39.5) | 1,203 (33.9) |  |
| 60- | 6,586 (26.6) | 1,136 (38.7) | 4,733 (28.8) | 717 (15.9) |  |
| **Gender** |  |  |  |  |  |
| Female | 10,094 (51.5) | 1,277 (53.3) | 6,397 (48.3) | 2,420 (59.1) | **< 0.001** |
| Male | 9,650 (48.5) | 1,161 (46.7) | 6,825 (51.7) | 1,664 (40.9) |  |
| **Race/ethnicity** |  |  |  |  |  |
| Non-Hispanic White | 9,071 (70.9) | 1,108 (68.2) | 6,029 (70.4) | 1,934 (73.3) | **< 0.001** |
| Non-Hispanic Black | 3,933 (9.8) | 681 (15.5) | 2,752 (10.5) | 500 (5.6) |  |
| Mexican American | 2,763 (7.5) | 305 (6.9) | 1,927 (7.8) | 531 (6.9) |  |
| Other races | 3,977 (11.8) | 344 (9.5) | 2,514 (11.2) | 1,119 (14.1) |  |
| **Education level** |  |  |  |  |  |
| Less than 9th grade | 1,607 (4.0) | 318 (8.1) | 1,107 (4.0) | 182 (2.3) | **< 0.001** |
| 9-11th grade (includes  12th grade with no diploma) | 2,563 (9.4) | 497 (17.1) | 1,807 (10.4) | 259 (4.0) |  |
| High school graduate/GED  or equivalent | 4,502 (22.6) | 687 (31.0) | 3,261 (25.3) | 554 (12.2) |  |
| Some college or AA degree | 6,041 (31.8) | 690 (31.5) | 4,173 (33.4) | 1,178 (27.6) |  |
| College graduate or above | 5,031 (32.1) | 246 (12.3) | 2,874 (26.9) | 1,911 (53.9) |  |
| **PIR** | 3.09 ± 0.04 | 2.41 ± 0.06 | 3.03 ± 0.04 | 3.53 ± 0.05 | **< 0.001** |
| <1.3 | 5,936 (19.7) | 1,055 (32.5) | 4,012 (20.1) | 869 (13.4) | **< 0.001** |
| 1.3-3.5 | 7,464 (35.3) | 960 (40.6) | 5,133 (36.7) | 1,371 (29.5) |  |
| >3.5 | 6,344 (45.0) | 423 (27.0) | 4,077 (43.2) | 1,844 (57.0) |  |
| **Marital status** |  |  |  |  |  |
| Never married | 3,550 (17.5) | 308 (12.3) | 2,165 (15.8) | 1,077 (24.0) | **< 0.001** |
| Widowed/Divorced/Separated | 4,295 (17.9) | 792 (27.2) | 3,028 (19.5) | 475 (9.8) |  |
| Married/Living with partner | 11,899 (64.6) | 1,338 (60.4) | 8,029 (64.6) | 2,532 (66.2) |  |
| **Alcohol consumption** |  |  |  |  |  |
| Never | 2,593 (10.1) | 266 (8.9) | 1,699 (9.7) | 628 (11.4) | **< 0.001** |
| Former | 3,127 (12.9) | 668 (25.1) | 2,145 (13.4) | 314 (6.7) |  |
| Current | 14,024 (77.0) | 1,504 (66.0) | 9,378 (76.9) | 3,142 (81.9) |  |
| **Hypertension** |  |  |  |  |  |
| No | 11,349 (62.3) | 643 (29.6) | 7,174 (57.4) | 3,532 (88.3) | **< 0.001** |
| Yes | 8,395 (37.7) | 1,795 (70.4) | 6,048 (42.6) | 552 (11.7) |  |
| **COPD** |  |  |  |  |  |
| No | 18,743 (95.2) | 2,155 (88.5) | 12,562 (94.9) | 4,026 (98.6) | **< 0.001** |
| Yes | 1,001 (4.8) | 283 (11.5) | 660 (5.1) | 58 (1.4) |  |
| **DM** |  |  |  |  |  |
| DM | 3,283 (12.7) | 1,074 (39.7) | 2,132 (12.8) | 77 (1.6) | **< 0.001** |
| IFG | 954 (4.9) | 143 (6.9) | 710 (5.7) | 101 (2.2) |  |
| IGT | 800 (3.6) | 92 (3.8) | 587 (4.1) | 121 (2.3) |  |
| No | 14,707 (78.8) | 1,129 (49.6) | 9,793 (77.5) | 3,785 (93.9) |  |
| **Chronic bronchitis** |  |  |  |  |  |
| No | 18,579 (94.3) | 2,140 (88.1) | 12,443 (94.0) | 3,996 (97.7) | **< 0.001** |
| Yes | 1,165 (5.7) | 298 (11.9) | 779 (6.0) | 88 (2.3) |  |
| **Asthma** |  |  |  |  |  |
| No | 16,856 (85.3) | 1,925 (78.5) | 11,345 (85.7) | 3,586 (87.1) | **< 0.001** |
| Yes | 2,888 (14.7) | 513 (21.5) | 1,877 (14.3) | 498 (12.9) |  |
| **CVD** |  |  |  |  |  |
| No | 17,624 (91.6) | 1,857 (78.5) | 11,809 (91.4) | 3,958 (97.5) | **< 0.001** |
| Yes | 2,120 (8.4) | 581 (21.5) | 1413 (8.6) | 126 (2.5) |  |
| **LE8 metric scores** |  |  |  |  |  |
| Overall | 68.76 ± 0.26 | 41.71 ± 0.17 | 65.96 ± 0.12 | 86.86 ± 0.13 | **< 0.001** |
| Diet | 39.36 ± 0.55 | 19.03 ± 0.61 | 34.98 ± 0.52 | 58.99 ± 0.74 | **< 0.001** |
| Physical activity | 74.35 ± 0.50 | 26.65 ± 1.26 | 73.39 ± 0.56 | 95.79 ± 0.29 | **< 0.001** |
| Nicotine exposure | 72.21 ± 0.54 | 42.10 ± 1.26 | 69.21 ± 0.58 | 92.05 ± 0.47 | **< 0.001** |
| Sleep health | 83.73 ± 0.31 | 66.24 ± 0.88 | 82.95 ± 0.29 | 92.70 ± 0.33 | **< 0.001** |
| Body mass index | 60.10 ± 0.45 | 31.01 ± 0.82 | 55.07 ± 0.43 | 84.92 ± 0.48 | **< 0.001** |
| Blood lipids | 64.72 ± 0.37 | 43.09 ± 0.86 | 60.93 ± 0.44 | 83.29 ± 0.51 | **< 0.001** |
| Blood glucose | 85.74 ± 0.27 | 60.16 ± 0.70 | 85.10 ± 0.29 | 97.57 ± 0.21 | **< 0.001** |
| Blood pressure | 69.86 ± 0.39 | 45.36 ± 0.80 | 66.06 ± 0.41 | 89.57 ± 0.44 | **< 0.001** |
| Footnotes: Continuous variables are presented as mean ± SE, and categorical variables are presented as n (weighted %). ^a^ *P*-values were assessed by One-way ANOVA (continuous variables) or by Chi-square test (categorical variables). *P*-values shown in bold were statistically significant. Abbreviations: AA, Associate's Degree; COPD, Chronic obstructive pulmonary disease; CVD, Cardiovascular disease; CVH, Cardiovascular health; DM, Diabetes mellitus; GED, General educational development; IFG, Impaired fasting glycaemia; IGT, Impaired glucose tolerance; LE8, Life’s Essential 8; NHANES, National Health and Nutrition Examination Survey; PIR, Poverty income ratio; SE, Standard error. | | | | | |

| **Table S4 Association of LE8 scores with COPD for additional adjustments, NHANES 2007-2018 (n = 19,744).** | | | | | | | | | |
| --- | --- | --- | --- | --- | --- | --- | --- | --- | --- |
|  | **LE8 score** | | | | | | | | |
|  | **Low CVH (0-49)** |  | **Moderate CVH (50-79)** | |  | **High CVH (80-100)** | |  | ***P* for trend** |
|  |  |  | **AOR (95% CI)** | ***P*-value** |  | **AOR (95% CI)** | ***P*-value** |  |  |
| **Original** | Reference |  | **0.477 (0.394,0.578)** | **< 0.001** |  | **0.169 (0.115,0.249)** | **< 0.001** |  | **< 0.001** |
| **Adding** |  |  |  |  |  |  |  |  |  |
| **Survey cycle** | Reference |  | **0.518 (0.428, 0.626)** | **< 0.001** |  | **0.198 (0.139, 0.283)** | **< 0.001** |  | **< 0.001** |
| **DM** | Reference |  | **0.526 (0.437, 0.634)** | **< 0.001** |  | **0.204 (0.140, 0.298)** | **< 0.001** |  | **< 0.001** |
| **Hypertension** | Reference |  | **0.520 (0.432, 0.625)** | **< 0.001** |  | **0.197 (0.135, 0.287)** | **< 0.001** |  | **< 0.001** |
| **CVD** | Reference |  | **0.551 (0.460, 0.659)** | **< 0.001** |  | **0.215 (0.151, 0.307)** | **< 0.001** |  | **< 0.001** |
| Footnotes: The multivariable logistic regression model was adjusted for age, gender, race/ethnicity, education level, marital status, PIR, and alcohol consumption. Survey cycle, DM (yes or no), hypertension (yes or no), and CVD (yes or no) were added into the multivariable logistic regression model for additional adjustment, respectively. Results of AOR (95% CI), *P* for trend, and *P*-value presented with bold values were statistically significant with *P*-value < 0.05 or *P*-value < 0.001.  Abbreviations: AOR, Adjusted odds ratio; CI, Confidence interval; COPD, chronic obstructive pulmonary disease; CVD, Cardiovascular disease; CVH, Cardiovascular health; DM, Diabetes mellitus; LE8, Life’s Essential 8; NHANES, National Health and Nutrition Examination Survey; PIR, Poverty income ratio. | | | | | | | | | |

| **Table S5 Association of the LE8 score with COPD, excluding 277 underweight participants.** | | | | | | | | |
| --- | --- | --- | --- | --- | --- | --- | --- | --- |
|  | **Crude model** | |  | **Model 1** | |  | **Model 2** | |
|  | **COR (95% CI)** | ***P*-value** |  | **AOR (95% CI)** | ***P*-value** |  | **AOR (95% CI)** | ***P*-value** |
| **LE8 score** |  |  |  |  |  |  |  |  |
| Low CVH (0-49) | Reference | - |  | Reference | - |  | Reference | - |
| Moderate CVH (50-79) | **0.410 (0.346, 0.485)** | **< 0.001** |  | **0.449 (0.377, 0.535)** | **< 0.001** |  | **0.510 (0.428, 0.607)** | **< 0.001** |
| High CVH (80-100) | **0.110 (0.076, 0.159)** | **< 0.001** |  | **0.158 (0.110, 0.228)** | **< 0.001** |  | **0.199 (0.138, 0.287)** | **< 0.001** |
| *P* for trend |  | **< 0.001** |  |  | **< 0.001** |  |  | **< 0.001** |
| Footnotes: The crude model was unadjusted. Model 1 was adjusted for age, gender, and race/ethnicity. Model 2 was additionally adjusted for education level, marital status, PIR, and alcohol consumption. The results of COR (95% CI), AOR (95% CI), and *P*-value shown in bold were statistically significant. *P*-value < 0.05 or *P*-value < 0.001.  Abbreviations: AOR, Adjusted odds ratio; CI, Confidence interval; COPD, Chronic obstructive pulmonary disease; COR, Crude odds ratio; CVH, Cardiovascular health; LE8, Life’s Essential 8; NHANES, National Health and Nutrition Examination Survey; PIR, Poverty income ratio. | | | | | | | | |

| **Table S6 Association of the LE8 score with COPD, excluding 2,888 patients with asthma.** | | | | | | | | |
| --- | --- | --- | --- | --- | --- | --- | --- | --- |
|  | **Crude model** | |  | **Model 1** | |  | **Model 2** | |
|  | **COR (95% CI)** | ***P*-value** |  | **AOR (95% CI)** | ***P*-value** |  | **AOR (95% CI)** | ***P*-value** |
| **LE8 score** |  |  |  |  |  |  |  |  |
| Low CVH (0-49) | Reference | - |  | Reference | - |  | Reference | - |
| Moderate CVH (50-79) | **0.489 (0.383, 0.626)** | **< 0.001** |  | **0.466 (0.358, 0.606)** | **< 0.001** |  | **0.528 (0.400, 0.698)** | **< 0.001** |
| High CVH (80-100) | **0.101 (0.065, 0.157)** | **< 0.001** |  | **0.113 (0.071, 0.181)** | **< 0.001** |  | **0.150 (0.094, 0.238)** | **< 0.001** |
| *P* for trend |  | **< 0.001** |  |  | **< 0.001** |  |  | **< 0.001** |
| Footnotes: The crude model was unadjusted. Model 1 was adjusted for age, gender, race/ethnicity, and obesity. Model 2 was additionally adjusted for education level, marital status, PIR, and alcohol consumption. The results of COR (95% CI), AOR (95% CI), and *P*-value shown in bold were statistically significant. *P*-value < 0.05 or *P*-value < 0.001.  Abbreviations: AOR, Adjusted odds ratio; CI, Confidence interval; COPD, Chronic obstructive pulmonary disease; COR, Crude odds ratio; CVH, Cardiovascular health; LE8, Life’s Essential 8; PIR, Poverty income ratio. | | | | | | | | |
